# Biochemical fluctuations, optimisation and the linear noise approximation

**DOI:** 10.1186/1752-0509-6-86

**Published:** 2012-07-17

**Authors:** Jürgen Pahle, Joseph D Challenger, Pedro Mendes, Alan J McKane

**Affiliations:** 1 School of Computer Science and Manchester Centre for Integrative Systems Biology, The University of Manchester, 131 Princess Street, Manchester M1 7DN, UK; 2 Theoretical Physics Division, School of Physics and Astronomy, The University of Manchester, Manchester, M13 9PL, UK; 3 Virginia Bioinformatics Institute, Virginia Tech, Washington Street 0477, Blacksburg, VA 24061, USA

**Keywords:** Linear noise approximation, Optimisation, Mitogen-activated kinases signalling, COPASI, Intrinsic noise, Stochastic biochemical models, Systems biology

## Abstract

**Background:**

Stochastic fluctuations in molecular numbers have been in many cases shown to be crucial for the understanding of biochemical systems. However, the systematic study of these fluctuations is severely hindered by the high computational demand of stochastic simulation algorithms. This is particularly problematic when, as is often the case, some or many model parameters are not well known. Here, we propose a solution to this problem, namely a combination of the linear noise approximation with optimisation methods. The linear noise approximation is used to efficiently estimate the covariances of particle numbers in the system. Combining it with optimisation methods in a closed-loop to find extrema of covariances within a possibly high-dimensional parameter space allows us to answer various questions. Examples are, what is the lowest amplitude of stochastic fluctuations possible within given parameter ranges? Or, which specific changes of parameter values lead to the increase of the correlation between certain chemical species? Unlike stochastic simulation methods, this has no requirement for small numbers of molecules and thus can be applied to cases where stochastic simulation is prohibitive.

**Results:**

We implemented our strategy in the software COPASI and show its applicability on two different models of mitogen-activated kinases (MAPK) signalling -- one generic model of extracellular signal-regulated kinases (ERK) and one model of signalling via p38 MAPK. Using our method we were able to quickly find local maxima of covariances between particle numbers in the ERK model depending on the activities of phospho-MKKK and its corresponding phosphatase. With the p38 MAPK model our method was able to efficiently find conditions under which the coefficient of variation of the output of the signalling system, namely the particle number of Hsp27, could be minimised. We also investigated correlations between the two parallel signalling branches (MKK3 and MKK6) in this model.

**Conclusions:**

Our strategy is a practical method for the efficient investigation of fluctuations in biochemical models even when some or many of the model parameters have not yet been fully characterised.

## Background

Random fluctuations in discrete molecular numbers can have significant impact, both detrimental and constructive, on the functioning of biochemical systems [1,2]. Systems that contain only relatively small numbers of particles of a certain chemical species, such as in signal transduction or gene expression, are particularly prone to this intrinsic noise. Here, the underlying discreteness of the system and stochastic timing of reactive events can lead to fluctuations in species abundances of high relative amplitude. Even when particle numbers are high, stochastic effects can significantly affect the dynamic behaviour of certain biochemical networks [3].

Biochemical systems have evolved to be robust against molecular fluctuations by attenuation, or even to exploit them (see [4,5] for examples). Therefore, these fluctuations should be considered whenever quantitative and dynamic models are devised to describe biochemical systems. Different mathematical formalisms have been developed to allow stochastic modelling and to explicitly take into account random fluctuations. Such systems are usually modelled by a continuous-time Markov process which follows the chemical master equation. The chemical master equation describes the time evolution of the system state probability distribution, *i.e. *how probable it is that a chemical species in the system will have specific particle numbers at a specific point in time. Both analytic and numerical solutions of this chemical master equation are difficult to obtain for most biologically relevant systems. Even though there exist methods to numerically solve the master equation [6] these are only feasible for relatively simple systems. A popular substitute is to apply Gillespie's stochastic simulation algorithm [7] to calculate single trajectories of the system's dynamics. By calculating very many of such (random) instances one can then approximate the trajectory of the probability density function of each chemical species and calculate relevant time-dependent statistics, such as the mean value or covariances. However, the stochastic simulation of single trajectories alone can be computationally demanding. The calculation of very many of them quickly becomes impracticable even when accelerated approximate stochastic simulation methods [8] are employed.

For a quick characterisation of the fluctuations in a biochemical system there exists an alternative, namely the linear noise approximation (LNA; see, *e.g*., [9-11]). This approximate method is based on van Kampen's system-size expansion of the chemical master equation [12-14]. The LNA estimates the variances of the species abundances and the covariances between them. Even though, theoretically, the LNA is only locally valid in the vicinity of macroscopic steady states or other system trajectories, in practical terms, it often gives good results even when the behaviour of the stochastic model and the behaviour of the corresponding deterministic model are quite different [15]. The LNA is particularly interesting because it is independent of computationally demanding stochastic simulations but, instead, only uses information about the stoichiometries in the system and the macroscopic reaction rates -- therefore it can be calculated very quickly. Other approaches have also been proposed for the estimation of steady state noise. For instance, in [16], analytical estimates of the fluctuations are found using error growth techniques. These are based on ideas from nonlinear dynamics and do not begin from a master equation. This is in contrast to the work presented here, where the molecular basis of the model is central, and where the nature of the fluctuations can be explicitly calculated. There are, of course, many studies of fluctuations in biochemical systems. For instance, in [17] the authors use data from time series to infer the values of the model parameters. This is in some sense the converse of our approach.

Often, in practice, one or more of the parameters of a model, such as reaction rates or initial concentrations, cannot be exactly determined. For instance, such parameters might only be known to lie within a certain range or nothing might be known about them at all. This uncertainty about parameters can translate into uncertainty about the system behaviour when it has high sensitivity towards those parameters. This is also true for molecular fluctuations in the system since their expected amplitude and other properties depend on parameter values. If only one or two parameters are unknown it is possible to exhaustively scan this parameter space using a regular grid or other techniques to probe how the model is affected by variations in values of those parameters. However, this approach is not feasible if the number of unknown parameters is large since the hyper-volume of the parameter search space increases exponentially with the number of uncertain parameters, and consequently so does the computational time.

In this article we introduce a different strategy to study random fluctuations in biochemical models with parameters that are not well characterised. Our approach combines the LNA with optimisation methods to search the unknown parameter space for parameter values that lead to extrema in covariance estimates. This can dramatically reduce the required computation time compared to exhaustive searches with stochastic simulations, thereby permitting types of studies of stochastic fluctuations that were not possible before. We will show a relevant biological example of a search for conditions that minimise the noise in the output of a p38 MAPK signalling system. Scanning the parameter space and using stochastic simulation is clearly impossible here because this would take more than 2.4 · 10^17 ^years. Our method, in contrast, was able to find these conditions in 25 min. Therefore, the strategy we are proposing makes it possible to gain biological insight about the noise structure of relevant biological systems even if these systems are big and the parameters are not well defined.

Global optimisation methods have been shown to be effective in finding good extrema estimates of dynamic properties of biochemical network models even in high-dimensional search spaces [18,19]. The strategy proposed here is similar to an earlier one successfully applied to the search for extreme values of sensitivities [20].

The application of this strategy passes through a closed loop containing the automatic calculation of a steady state, the LNA method and one optimisation algorithm; alternatively the method is also appropriate to use with parameter scanning or sampling algorithms instead of the optimisation. We implemented this strategy in the software COPASI [21,22], which already contains optimisation, scanning and sampling algorithms. We demonstrate the application of this new strategy on two different models of mitogen-activated kinase (MAPK) signalling pathways, namely a model of extracellular signal-regulated kinases (ERK) by Kholodenko [23] and a model of p38 MAPK by Hendriks *et al. *[24].

## Results

### Implementation of the method in COPASI

**Figure 1:**
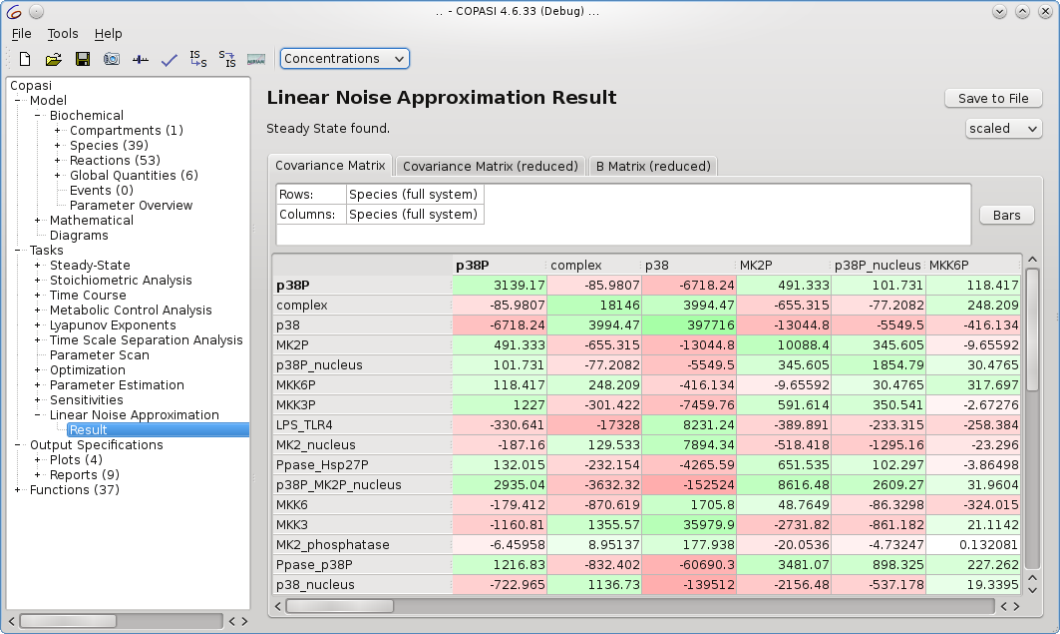
**Screen shot of the LNA implementation in COPASI**. Screen shot of the COPASI graphical user interface and the linear noise approximation task. Shown is the resulting covariance matrix of species' particle numbers in the p38 MAPK model by Hendriks *et al. *[24]. The matrix is colour coded, positive values have a green background and negative values a red one with intensities corresponding to the absolute values.

The software COPASI [21,22] gives all interested researchers easy access to modelling and simulation for biochemical networks, because it is freely available under the Artistic license version 2.0 at [22] and supports the Systems Biology Markup Language (SBML) standard [25] for the exchange of model files with other software. An implementation of the method described here was integrated in COPASI, comprising a new LNA task that, using the linear noise approximation (see Methods), generates as output a matrix of covariance estimates between all the species' particle numbers in a given biochemical model (see Figure 1). Prior to this, the method can also automatically calculate a steady state for the model which is important if parameters, and thus the system's steady state, have been changed. The covariances estimated by the LNA task can then be subsequently used by other tasks in COPASI, in particular optimisation, parameter scanning or sampling in a closed-loop fashion. This combination results in a practical method for the investigation of fluctuations in models even when some or many of the model parameters have not yet been fully characterised.

Our implementation allows arbitrary objective functions to be optimised. For instance, LNA estimates of covariances of different chemical species, as well as other model observables, can be combined into a complex objective function. This allows the calculation of various quantities of interest, for instance, Fano factors [26] or coefficients of variation (CV), as shown below. In terms of parameter search, our implementation can use a large variety of numerical optimisation algorithms, both local and global, that are accessible in COPASI -- gradient-based, particle swarm [27], simulated annealing, evolutionary algorithms and others [28]. This is particularly important since the performance of global optimisation algorithms has been shown to be problem-dependent, and no single one is guaranteed to converge to a global optimum for all problems [29].

### Application of the method on MAPK signalling systems

Signalling through mitogen-activated protein kinases (MAPK) is involved in a broad range of cellular processes, such as proliferation, differentiation, stress responses and apoptosis. Therefore it is also implicated in a variety of diseases like cancer, stroke or diabetes [30]. As such, it has been the object of a number of computational modelling studies that helped elucidate dynamic properties of the system, such as amplification of signals, noise reduction or switching behaviour [31].

There exist different specific MAPK signalling pathways with different functions, for example ERK1/2, p38 or JNK, with different topologies and characteristics. However, in most cases the basic structure is that of a three-tier cascade. Here, the MAPKs on the output level, such as ERK1/2 or p38, phosphorylate transcription factors or other proteins to trigger specific cellular responses. The MAPKs are, in turn, activated *via *phosphorylation by other protein kinases, so-called MAP2K (or MKK) that are themselves activated by MAP3K (or MKKK) further upstream.

### Fluctuations in a model of ultrasensitivity in ERK MAP kinase signalling

**Figure 2:**
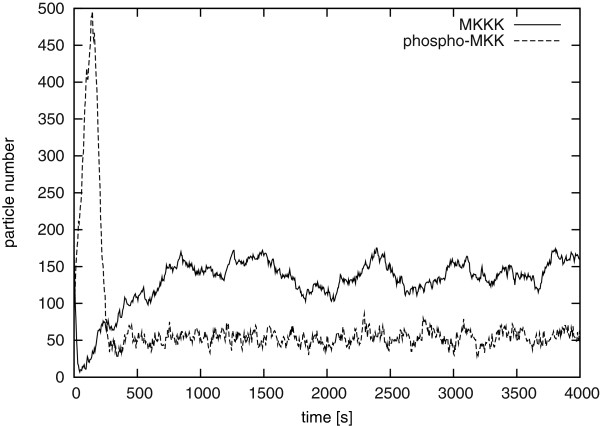
**Stochastic simulation of the ERK MAPK model**. MKKK and phospho-MKK particle numbers *vs. *time [s], with *K*_*I *_= 45, *V_cell _*= 10^-14 ^l and all other parameters as in [23].

We will now apply the LNA to a MAPK cascade model due to Kholodenko [23], which is a popular model of a generic extracellular signal-regulated kinases (ERK) MAPK signalling cascade. Due to a negative feedback loop, the model can exhibit limit cycle behaviour for some parameter values, and a stable steady-state for others. While Kholodenko examined the model in the limit cycle regime [23], we reduced the feedback strength by increasing the kinetic constant *K_I _*to 45, so that a stable steady-state exists (all other parameter values remain as in the original paper). A typical stochastic simulation of the system is shown in Figure 2, simulated with Gillespie's Direct Method [7] (as implemented in COPASI).

It is interesting to see how the magnitude of fluctuations changes with the reaction parameters. As an example, we used our LNA implementation in COPASI in combination with a parameter scan to investigate how changes in the reaction parameter *v*_2 _affect the variance of MKKK (MAPK kinase kinase). Values of *v*_2 _were scanned within a certain range and the LNA automatically calculated for each value of *v*_2_. In the model, this parameter corresponds to the *V_max _*of phospho-MKKK dephosphorylation and so refers to the activity of MKKK-phosphatase.

**Figure 3:**
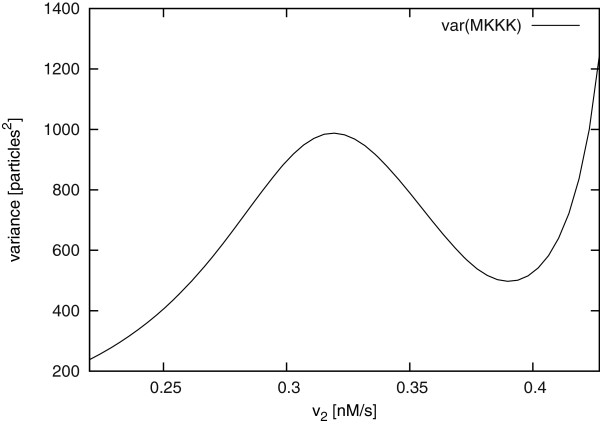
**Parameter scan of MKKK particle number variance against reaction parameter ***v*_2 _**in the ERK MAPK model**. A parameter scan of the variance of the particle number of species MKKK has been carried out for a range of values of the reaction parameter *v*_2_, with *K_I _*= 45, *V_cell _*= 10^-14 ^l and all other parameters as in [23].

Presently protein kinases are much better characterised at the molecular level than protein phosphatases. As a consequence the effect of phosphatases are often also not studied in signalling models. However, here we are able to show that the activity of the MKKK-phosphatase does not only influence the type of dynamics the system exhibits, namely that the steady state becomes unstable at *v*_2 _= 0.446 due to a Hopf bifurcation. It also strongly affects the intrinsic fluctuations in the system. As can be seen in Figure 3, the estimated variance of MKKK becomes large as *v*_2 _approaches the bifurcation point and, interestingly, it shows a local maximum at *v*_2 _= 0.32 of 987.7 particles^2^. The value of *v*_2 _in Figure 3 does not go as far as the bifurcation point, as the LNA loses accuracy near this value.

**Figure 4:**
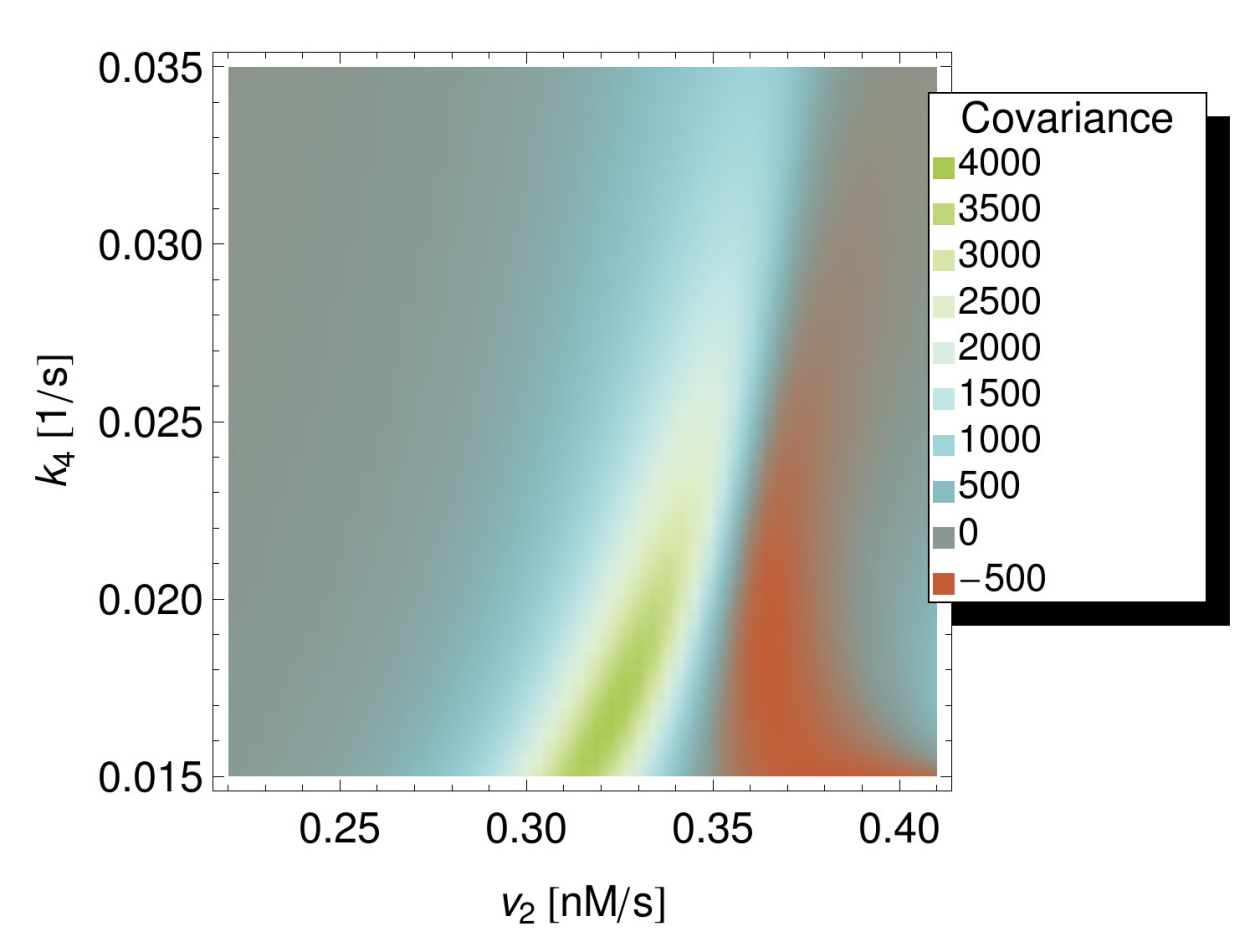
**Two-dimensional parameter scan of MKKK and MKK-P particle numbers' covariance in the ERK MAP model**. A two-dimensional parameter scan of the covariance of the particle numbers of species MKKK and MKK-P. The parameter *v*_2 _was varied between 0.22 and 0.41 and the parameter *k*_4 _between 0.015 and 0.035.

We then wanted to investigate the conditions under which fluctuations in chemical species at different positions of the signalling cascade become correlated. To achieve this, we used the optimisation task in COPASI to maximise the covariance of the fluctuations of MKKK and MKK-P, allowing the reaction parameters *v*_2 _and *k*_4 _to vary over a given range of values. Using the evolutionary programming algorithm [28] (which took 199 seconds to run) 4004 steady state and LNA evaluations were carried out. A local maximum of the covariance was found with a value of 4035 particles^2 ^for *v*_2 _= 0.3226 and *k*_4 _= 0.0166. The algorithm converged to this value already after 880 iterations. A parameter scan over the same parameter space was also performed to better illustrate the change in correlation with these two parameters. Figure 4 shows how the covariance of the fluctuations of MKKK and MKK-P varies with the reaction parameters, and provides a visualisation of the landscape that the optimisation algorithm must traverse. Note that the covariance becomes negative for some parameter values.

### Fluctuations in a model of p38 MAPK signalling

The so-called p38 mitogen-activated protein kinases (p38 MAPK) are responsive to proinflammatory cytokines and stress factors [32]. One prominent signal are lipopolysaccharides (LPS), which are components in the cell wall of bacteria. Their presence indicates a bacterial infection and triggers a strong immune response in animals. The MAPK of this pathway, p38, can, *inter alia*, activate MAP kinase-activated protein kinase 2 (MK2). One substrate of MK2 is the heat shock protein 27 (Hsp27) and the concentration of the active/phosphorylated form of Hsp27 is regularly used to estimate the activity of the p38 MAPK signalling pathway. The level of Hsp27 will also represent the main signalling output in the model.

The model we use for this study was developed in Hendriks *et al. *2008 [24]. Its structure is shown in the additional file 1. The original model included the rapid inactivation of a (TAK1:TAB1:TAB2) complex. This was represented by a degradation reaction which, after an initial peak, led to an abrogation of p38 MAPK activity. For this study we removed this degradation reaction which allows the system to reach a steady state of sustained p38 MAPK signalling depending on the amount of LPS. We also reformulated the model in such a way that it no longer contained three compartments (medium, cytosol and nucleus) but, instead, uses a single reference volume for all species, including the nuclear ones, corresponding to the volume of the whole cell. This was needed because the current implementation of the LNA can only handle models with one compartment. In [24] the model was fitted to experimental measurements, and in the following we will use the set of parameters which showed the best fit.

As mentioned above, random fluctuations in signalling systems are particularly interesting to study, since here copy numbers of the different species are often low. For instance, MKK3 and MKK6 are typically present in the order of only ten thousand particles per cell. This could lead to pronounced fluctuations which hamper reliable information transfer through this signalling pathway. But perhaps there are conditions (parameter values) for which these fluctuations are minimised, which is what we want to investigate.

**Figure 5:**
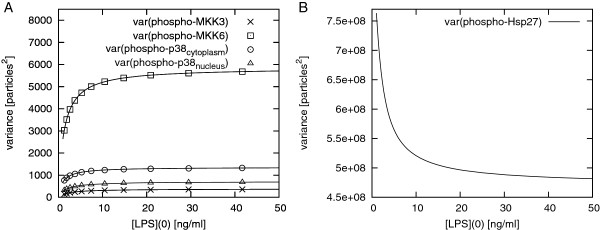
**Variances of species' particle numbers versus stimulation strength in the p38 MAPK model**. Panel A: Variance of cytosolic phospho-MKK3 (×), phospho-MKK6 (□), phospho-p38 (○), and nuclear phospho-p38 (Δ) particle numbers *vs. *strength of stimulation (concentration of LPS [ng/ml]). Panel B: Variance of cytosolic phospho-Hsp27 versus strength of stimulation (concentration of LPS [ng/ml]).

First we looked at the estimated variances of different signalling intermediates, such as phospho-MKK3, phospho-MKK6, cytosolic phospho-p38 and nuclear phospho-p38 with varying stimulus strength, *i.e. *concentration of LPS (Figure 5, panel A). We performed a parameter scan in COPASI where the initial concentration of LPS was varied within a certain range and the LNA was automatically calculated at each LPS concentration. We found that the variances increase with increasing stimulus strength but saturate at high values of LPS (resembling hyperbolic functions).

By contrast, phospho-Hsp27, the endpoint of the modelled signalling pathway, shows a decrease in its variance with increasing stimulation (Figure 5, panel B).

**Figure 6:**
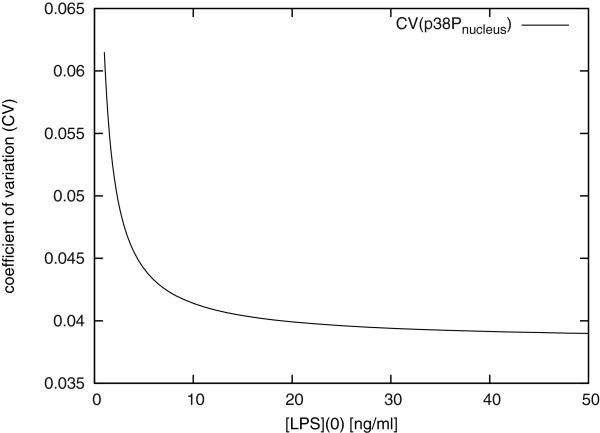
**Coefficient of variation of nuclear phospho-p38 *vs. *stimulation strength in the MAPK model**. Coefficient of variation of nuclear phospho-p38 *vs. *concentration of LPS [ng/ml].

However, looking at the coefficient of variation (CV) both nuclear phospho-p38 and cytosolic phospho-Hsp27 show a decrease of variation with increasing stimulation due to increasing steady state particle numbers (Figure 6 shows the CV of nuclear phospho-p38 against the concentration of LPS). This means that, in both cases, the relative amplitude of fluctuations decreases with increasing signal strength -- the higher the stimulus, the less ambiguous it becomes.

An interesting property of the p38 MAPK pathway is the existence of two parallel signalling branches, through MKK3 and MKK6, that both can phosphorylate p38 MAPK. Therefore, we were interested in whether fluctuations in the MKK3 branch correlate with fluctuations in the MKK6 branch. First, we scanned the estimated covariance of phospho-MKK3 and phospho-MKK6 over a range of stimulus strengths. We found that the fluctuations in the two branches seem to be mostly uncorrelated (the LNA actually estimates a very weak anti-correlation for higher initial concentrations of LPS, data not shown), an indication that the largest part of the fluctuations does not originate from the common upstream part of the two branches but rather from within the branches themselves.

We now wanted to investigate how the parameters in the system influence this anti-/correlation. Therefore, we searched for extreme values of the LNA-estimated correlation coefficient of phospho-MKK3 and phospho-MKK6 $\rho \;\left(\text{phospho-MKK}3,\;\text{phospho-MKK}6\right)=\frac{\text{cov}\left(\text{phospho-MKK}3,\text{phospho-MKK}6\right)}{\sqrt{\text{var(phospho-MKK3})}\;*\;\sqrt{\text{var}(\text{phospho-MKK}6)}}$ within a fixed, but large, range of all parameter values.

We therefore ran the LNA in combination with the particle swarm optimisation algorithm of COPASI, using the correlation coefficient as the objective function for maximisation. In addition we set constraints on the number of steady state particle numbers in the system. Both phospho-MKK3 and phospho-MKK6 particle numbers were allowed to change only 4-fold, *i.e. *within 50% - 200% of their original values. All other species' particle numbers were allowed to change 100-fold, *i.e. *within 10% - 1000% of their original values. The reasons for this were, firstly, that we did not primarily want to change the steady state of the system but rather only wanted to affect the fluctuations around the steady state. Secondly, if particle numbers are not constrained the optimisation often converges towards degenerate cases where one or both of the steady state particle numbers are very close to zero, *i.e. *the lower limit -- a situation where the LNA estimation can have large errors due to its assumption of Gaussian fluctuations.

We used a particle swarm optimisation [27] method with a swarm size of 50. The parameters to vary were all 29 reaction rates of the first 20 reactions in the model (see [24]), which includes all receptor (complex)-related reactions, both MKK3 and MKK6 branches, and the phosphorylation and dephosphorylation of p38. The parameters were allowed to change 100-fold, *e.g. *within 10% - 1000% of their original values. With these settings our method was able to find conditions where the estimated correlation between phospho-MKK3 and phospho-MKK6 was larger than 0.95 with a computation time of roughly 70 min.

Finally, we were interested in the influence that different choices for parameters in the two branches have on the fluctuations of the output of the signalling pathway (phospho-Hsp27) or, in other words, how reliable or noisy the overall signalling pathway can be. We used a particle swarm optimisation (swarm size = 50) [27] in combination with the LNA to minimise the coefficient of variation (CV) of phospho-Hsp27 $\left(\text{CV}\left(\text{phospho-Hsp27}\right)=\frac{\sqrt{\text{var(phospho-Hsp27)}}}{\left|\text{phospho-Hsp27}\right|}\right)$. The parameters that were allowed to change were the 21 reaction rates of all reactions listed in Table 1. All rates were allowed to change 4-fold (*e.g. *from 50% to 200% of their original value). Column "Changes (no constraints)" in Table 1 details how the optimisation minimised CV(phospho-Hsp27). Most of the rates were increased or decreased until they reached the given limits. Briefly, one can see that the phosphorylation steps of MKK3, MKK6 and p38 are made faster, whereas their respective dephosphorylations are made slower. Obviously, the CV can be minimised by just increasing the steady state particle number and leaving the variance as it is. Because this was the result of our first attempt, we carried out a second run where we constrained the phospho-Hsp27 particle number to stay below the limit of 4.65 · 10^6 ^particles. With the original parameter set the steady-state particle number of phospho-Hsp27 was 4.647 · 10^6 ^particles. The result of this second calculation is shown in column "Changes (constrained)" of Table 1. The most notable differences compared to the unconstrained case can be found in the MKK3 branch. Now the phosphorylation of p38 by phospho-MKK3 (MKK3P) is slower than in the original model. Also, the rates for both the binding and dissociation of phospho-p38 (p38P) and its phosphatase (p38_phosphatase) have been increased as well as the rate for the corresponding dephosphorylation. The binding of phospho-MKK6 and its phosphatase (MKK6_phosphatase) is now faster while the rate for the corresponding dissociation seems to be almost unchanged from its original value.

**Table 1:** Optimisation of the coefficient of variation of phospho-Hsp27 particle numbers

Reactions	Changes (no constraints)	Changes (constraints)
complex + MKK6 ↔ complex_MKK6	⇒	⇒
complex_MKK6 → complex + MKK6P	⇒	⇒
MKK6_phosphatase + MKK6P ↔ Ppase_MKK6P	⇐	~
Ppase_MKK6P → MKK6_phosphatase + MKK6	⇐	⇐
complex + MKK3 ↔ complex_MKK3	⇒	⇒
complex_MKK3 → complex + MKK3P	⇒	⇒
MKK3_phosphatase + MKK3P ↔ Ppase_MKK3P	⇐	⇐
Ppase_MKK3P → MKK3_phosphatase + MKK3	⇐	⇐
MKK6P + p38 ↔ MKK6P_p38	⇒	⇒
MKK6P_p38 → MKK6P + p38P	⇒	⇒
MKK3P + p38 ↔ MKK3P_p38	⇒	⇐
MKK3P_p38 → MKK3P + p38P	⇒	⇐
p38_phosphatase + p38P ↔ Ppase_38P	⇐	⇔
Ppase_p38P → p38_phosphatase + p38	⇐	⇒

Optimisation of the coefficient of variation of phospho-Hsp27 particle numbers with regards to all 21 reaction parameters of the listed reactions ([LPS]_0 _= 1 ng/ml). "Changes (no constraints)" means that the coefficient was optimised without any further constraints, whereas "Changes (constrained)" means that during optimisation the phospho-Hsp27 particle number was constrained in the optimisation to stay below the limit of 4.65 million particles. "⇒" ("⇐") denotes an increase (decrease) in the forward rate and a decrease (increase) in the reverse rate, in case of a reversible reaction. "⇔" means that both forward and reaction rates are increased and "~" means that the optimisation led to no clear change

We would like to note here that a (naive) comprehensive search for optima using a regular grid approach and stochastic simulations of the system in this particular case would have taken a prohibitively long computation time. Assuming that, within the given limits, we only look at ten different values per parameter we would have 10^<no.parameters>^= 10^21 ^sample points. For each point we would need to carry out a stochastic simulation that, including the calculation to allow the system to settle down to a steady state, takes approximately 7700 s on a typical desktop computer (for a simulated time of 10000 s). Neglecting the time needed to calculate the actual statistics on the simulated time series this would lead to a computation time of more than 10^21 ^· 7700 s ≈ 2.4 · 10^17 ^years. And this would only explore ten values of each parameter (*i.e. *it would be at a low resolution.) In contrast our method, using the linear noise approximation in combination with numerical optimisation, took 25 min to converge. This clearly shows the utility of the method we propose here: it makes tractable to calculate many phenomena that otherwise would be computationally prohibitive. Finally, although an approximation had to be adopted, it is typically so good that this has very little impact on the accuracy of the method.

## Discussion

Our contribution with this work is two-fold. First, we implemented the linear noise approximation in the freely available software COPASI, and thus made it accessible to a large group of users. Secondly, we showed how the LNA in combination with multi-dimensional parameter scans or with global numerical optimisation methods is appropriate to quickly characterise the influence of parameters on intrinsic fluctuations in biochemical models even when there is considerable uncertainty about a number of parameters. We showed, with realistic biochemical signalling models, that using this approach one is able to explore parameter space such that conditions can be found for which there is minimal, or maximal, noise. It is also possible to search for conditions where specific model variables are highly (or poorly) correlated. This new method thus provides a new and important way to explore the universe of behaviours displayed by models. Given the importance of noise and fluctuations in intracellular biochemistry, this method is therefore of great value for the study of those systems.

In the recent article by Komorowski *et al. *[33] a related method is proposed. There, the linear noise approximation is used to calculate Fisher information matrices for stochastic models, primarily to inform experimental design, *e.g. *by examining the information content of different experimental samples. Our approach, on the other hand, focuses on exploring the model independently of any physical measurements. Therefore, the two approaches are complementary.

In certain cases, however, care should be taken when using the LNA. This is due to the assumption that the fluctuations are Gaussian in nature. Problems can arise if the system is close to a boundary. For example, if the number of molecules for a particular species is very close to zero the probability distribution for the fluctuations becomes 'squashed' (which the LNA does not take into account), to satisfy the requirement that the probability to have a negative number of molecules present is zero. Boundaries can also arise due to conservation relations, which are discussed in the Methods section, as these add constraints to the system. When using the LNA in combination with one of the optimisation algorithms in COPASI, such systems near boundaries are sometimes found, especially when the user wishes to minimise a covariance, as we found when studying the p38 MAPK model. This is because the fluctuations can be very small when the system is close to a boundary, which can give the impression that the fluctuations of two different species are uncorrelated, which may not be the case away from the boundary. In these cases, adding constraints to the particle numbers (as we did when studying the p38 MAPK model) helps to keep the system away from these states. The current implementation of the LNA in COPASI is only able to consider models in which the reactions all occur within one compartment. As many biochemical models involve multiple compartments we hope to extend our work, so that in future it will be possible to use the LNA to study a wider range of models.

## Methods

Biochemical network models of the kind we analyse here can be described as consisting of $\hat{K}$ species $Y_{1},\ldots ,Y_{\hat{K}}$ enclosed in a volume *V*. There will be *M *reactions which interconvert species:

$$\begin{array}{ccc} r_{11}Y_{1}+\ldots +r_{\hat{K}1}Y_{\hat{K}} & \to & p_{11}Y_{1}+\ldots +p_{\hat{K}1}Y_{\hat{K}}\\ & \vdots & \\ r_{1M}Y_{1}+\ldots +r_{\hat{K}M}Y_{\hat{K}} & \to & p_{1M}Y_{1}+\ldots +p_{\hat{K}M}Y_{\hat{K}} \end{array}$$

where the numbers *r_iμ _*and *p_iμ _*$\left(i=1,\ldots ,\hat{K};\mu =1,\ldots ,M\right)$ describe respectively the population of the reactants and the products involved in the reaction. This may be written more compactly as

(1) $$\overset{\hat{K}}{\underset{i=1}{\sum }}r_{i\mu }Y_{i}\to \overset{\hat{K}}{\underset{i=1}{\sum }}p_{i\mu }Y_{i},\;\;\;\mu =1,2,\;\ldots M.$$

All the reactions above are strictly irreversible, therefore, without loss of generality, any chemically reversible reactions must be described as two separate irreversible reactions. The elements of the stoichiometry matrix, *ν_iμ _*≡ *p_iμ _*- *r_iμ_*, describe how many particles of species *Y_i _*are transformed due to the reaction *μ*. Although there are $\hat{K}$ species present in the system, they may not all be able to vary independently. This is because mass conservation relations are often present in the system which cause some variables to be linear combinations of others. As a simple example of this, consider the Michaelis-Menten reaction mechanism in an open system, described in Table 2.

**Table 2:** Michaelis-Menten reaction mechanism

Reactions	Kinetics
→ S	*v*_1 _= *k*_1_
S + E → SE	*v*_2 _= *k*_2 _· [S] · [E]
SE → S + E	*v*_3 _= *k*_3 _· [SE]
SE → P + E	*v*_4 _= *k*_4 _· [SE]
P →	*v*_5 _= *k*_5 _· [P]

A substrate, S, is converted to a product, P, *via *an enzyme E. The substrate and enzyme form a complex, SE. A constant flux of S molecules is supplied to the system and P molecules are able to leave the system. In our notation above, *Y*_1 _= S, *Y*_2 _= E, *Y*_3 _= P and *Y*_4 _= SE. Also, *r*_11 _= 1, *r*_21 _= 0 and so on. The total number of enzyme molecules, *i.e. *the number of free enzyme molecules plus the enzyme molecules bound in a complex, is fixed. If the number of SE molecules decreases by one, the number of E molecules increases by one. This conservation relation means that there is only one independent variable here, not two. In general, if the system contains Λ conservation relations, then the dimension of the system can be reduced from $\hat{K}$ to $K=\hat{K}-\Lambda$. It is necessary to reduce the size of the system in this way to facilitate the linear algebra to be done later. In the Michaelis-Menten system above, $\hat{K}=4\;$ and Λ = 1, so *K *= 3.

To specify the model, kinetic functions ${\tilde{f}}_{\mu }\left(n,V\right)$ associated with reaction *μ *need to be given. They are functions of the vector of particle numbers ***n ***= (*n*_1_, ...*, n_K_*) and volume *V*. Note that the vector of particle numbers has been 'shortened' from length $\hat{K}$ to *K *using the conservation relations. This will be discussed in more detail later in this section. In the limit where both the particle numbers and the volume become large, the kinetic functions become functions of the species concentration *n*_*i*_/*V *only; we then denote them by *f_μ_*(***x***), where *x_i _*= lim_*V*→∞ _n*_i_*/*V*. In this limit the conventional, macroscopic and deterministic, description of the systems applies and a set of ordinary differential equations (ODEs) can be written down to describe it:

(2) $$\frac{\text{d}x_{i}}{\text{d}t}=\overset{M}{\underset{\mu =1}{\sum }}\nu_{i\mu }f_{\mu }\left(x\right),\;\;\;i=1,\ldots ,K.$$

(The ODEs for the species that have been eliminated can be found by using the conservation relations.) However, the large system size limit is inappropriate for many systems of interest, in particular when the molecular populations are low (and the volume is small, as in most cells), then the discrete nature of the molecules has important consequences. In these cases a stochastic description is required.

The starting point for the stochastic description is the chemical master equation, which specifies how the probability that the system is in the state ***n ***at time *t*, *P*(***n***, *t*), changes with time. If *T_μ_*(***n***|***n*'**) is the transition rate from state ***n' ***to state ***n ***associated with reaction *μ*, then the master equation takes the form

(3) $$\frac{\text{d}P\left(n,t\right)}{\text{d}t}=\overset{M}{\underset{\mu =1}{\sum }}\left[T_{\mu }\left(n|n-\nu_{\mu }\right)P\left(n-\nu_{\mu },t\right)-T_{\mu }\left(n+\nu_{\mu }|n\right)P\left(n,t\right)\right],$$

where ***ν***_*μ *_= (*ν*_1*μ*_, ..., *ν_Kμ_*) is the stoichiometric vector corresponding to reaction *μ*. This completely defines the stochastic dynamics of the system once the initial condition *P*(***n***, 0) is given. If we multiply Eq. (3) by ***n***, and sum over all possible values of ***n ***one finds, after shifting the change of variable ***n ***→ ***n ***+ ***ν ***in the first term,

(4) $$\frac{\text{d}\langle n\left(t\right)\rangle }{\text{d}t}=\overset{M}{\underset{\mu =1}{\sum }}\nu_{\mu }\langle T_{\mu }\left(n+\nu_{\mu }|n\right)\rangle .$$

Dividing Eq. (4) by *V *and taking the limit *V *→ ∞, we see that we recover the deterministic description Eq. (2) if we make the identification *f_μ_*(***x***) = lim_*V*→∞_*V*^-1 ^〈*T_μ_*(***n ***+ ***ν***_*μ*_|***n***)〉. To go further than the macroscopic description Eq. (2) we need to develop an approximation scheme which goes beyond the deterministic dynamics in a systematic way. Fortunately such a scheme exists: the system-size expansion of van Kampen, which allows one to calculate corrections to the deterministic results in powers of *V*^-1*/*2 ^by writing $n/V=x+\xi /\sqrt{V}$, where ***x ***is found by solving Eq. (2). To next-to-leading order, which in general gives results in very good agreement with simulations, this is equivalent to assuming that the stochastic fluctuations are Gaussian, and so determined by stochastic processes which are linear. For this reason, this is frequently known as the linear noise approximation (LNA). Details of the general application of the method are given in the book by van Kampen [14], and for chemical reactions of the kind we are considering here by Elf and Ehrenberg [9]. In [14,34], terms an order smaller than the LNA are included. In most cases the LNA is very accurate, and these extra terms are not significant, provided the steady-state solution is not near a boundary, in which case the fluctuations would no longer be Gaussian. One finds that the stochastic dynamics of the LNA is governed by the Fokker-Planck equation

(5) ∂Π∂t=-∑i=1K∂∂ξiMiξΠ+12 ∑i,j=1KBij∂2 Π∂ξi∂ξj,

Where Π(ξ,t)=P(n,t) and $M_{i}\left(\xi \right)=\sum_{j=1}^{K}A_{ij}\xi_{j}$. Therefore the entire dynamics is defined by two matrices *A *and *B *which are given by

(6) $$A_{ij}\left(x\right)=\overset{M}{\underset{\mu =1}{\sum }}\nu_{i\mu }\frac{\partial f_{\mu }\left(x\right)}{\partial x_{j}},\;\;B_{ij}\left(x\right)=\overset{M}{\underset{\mu =1}{\sum }}\nu_{i\mu }\nu_{j\mu }f_{\mu }\left(x\right).$$

In all the investigations we will carry out in this paper, we will be interested in fluctuations about the stationary state. In terms of the deterministic dynamics Eq. (2), the solution ***x***(*t*) will be replaced by its fixed point value x*, and so the *A *and *B *matrices will be independent of time.

The Fokker-Planck Eq. (5) is linear, and so therefore its solution, Π(***ξ***, *t*), is Gaussian, and may be characterised by its first two moments. The ansatz used to set up the system-size expansion implies that the first moment, 〈*ξ_i_*(*t*)〉, is zero to this order. In the stationary state the covariance, 〈*ξ_i_*(*t*)*ξ_j_*(*t'*)〉 will only depend on |*t *- *t*'*|*. Therefore, Ξ*_ij _*≡ 〈*ξ_i_*(*t*)*ξ_j_*(*t*)〉 will be independent of time, and will satisfy [14]

(7) $$A\;\Xi +\;\Xi A^{\text{T}}+B=0.$$

All the matrices in Eq. (7) are dimension *K *× *K *and are independent of time. In applications we are frequently interested in the covariance in terms of particle numbers

(8) $$C_{ij}=\langle \left(n_{i}-\langle n_{i}\rangle \right)\;\left(n_{j}-\langle n_{j}\rangle \right)\rangle .$$

Since 〈*n_i_*〉 = *Vx_i _*and since the average of the fluctuations is zero to the order we are working,

(9) $$C_{ij}=\langle \left(\sqrt{V}\xi_{i}\right)\left(\sqrt{V}\xi_{j}\right)\rangle =V\Xi_{ij}.$$

The Lyapunov equation, analogous to Eq. (7) is therefore

(10) $$AC+CA^{\text{T}}+VB=0.$$

The equation can be solved for *C *numerically by employing the Bartels-Stewart algorithm [35]. Here, *A *is transformed to lower real Schur form and *A^T ^*is transformed to real Schur form. This allows elements of the transformed matrix *C *to be solved for successively. The solution of *C *is found by reversing the original transformation. It is important that the conservation relations are used to reduce the dimension of the system before the Bartels-Stewart algorithm is applied. The matrices for the 'unreduced' system will contain linearly dependent columns or, equivalently, zero eigenvalues. When this is the case, the solution to the equation is no longer unique [35].

Therefore our implementation of the LNA first automatically determines existing conservation relations (also known as conserved moieties) and reduces the system from $\hat{K}$ to *K *independent chemical species. Then the LNA is applied to the reduced system and the corresponding covariance matrix is calculated. In the last step the covariance matrix for the full system is recovered as follows.

For convenience, the state vector ***n ***should be written with the $K=\hat{K}-\Lambda$ independent species first *i.e. *filling the first *K *positions, and then the dependent species should be written at the end. This anticipates shortening ***n ***(when the size of the system is reduced) to contain *K *elements, rather than $\hat{K}$. The dependent species may be written in terms of the independent species by using the conservation equations which are linear combinations and so, in general, are of the form:

(11) $$n_{j}=c_{j}+\overset{K}{\underset{k=1}{\sum }}\alpha_{jk}n_{k},\;\;\;j=K+1,\ldots ,\hat{K},$$

where the *c_j _*and *α_jk _*are constants.

Examining the conservation relations after the change of variables used in the van Kampen expansion $\left(n_{j}\;=\;Vx_{j}\;+\;\sqrt{V}\xi_{j}\right)$ is introduced, the above equation becomes:

(12) $$Vx_{j}+\sqrt{V}\xi_{j}=c_{j}+\overset{K}{\underset{k=1}{\sum }}\alpha_{jk}\left(Vx_{k}+\sqrt{V}\xi_{k}\right).$$

But the conservation equations should hold in the deterministic limit (*V *→ ∞), too, *i.e*.

(13) $$Vx_{j}=c_{j}+\overset{K}{\underset{k=1}{\sum }}\alpha_{jk}Vx_{k}.$$

Therefore,

(14) $$\xi_{j}=\overset{K}{\underset{k=1}{\sum }}\alpha_{jk}\xi_{k}.$$

We can now use the above results to compute the remaining covariances. First of all we calculate Ξ*_ij_*, where *i *is an independent species and *j *is a dependent species:

(15) $$\Xi_{ij}=\langle \xi_{i}\xi_{j}\rangle =\langle \xi_{i}\left(\overset{K}{\underset{k=1}{\sum }}\alpha_{jk}\xi_{k}\right)\rangle ,$$

since 〈ξ*_i_*〉 = 〈ξ*_j_*〉 = 0. Now we have an expression for Ξ*_ij _*in terms of known quantities, the covariances of the independent species, which are found from solving the Lyapunov equation. Now we calculate Ξ*_ij _*for the case where *i *and *j *are both dependent species.

(16) $$\Xi_{ij}=\langle \xi_{i}\xi_{j}\rangle =\langle \left(\overset{K}{\underset{k=1}{\sum }}\alpha_{ik}\xi_{k}\right)\;\left(\overset{K}{\underset{l=1}{\sum }}\alpha_{jl}\xi_{l}\right)\rangle .$$

Again, we have obtained an expression in terms of known quantities. As before, *C_ij _*= *V*Ξ*_ij_*. Using a so-called link matrix *L *that connects the reduced and the full systems, as defined by Reder [36]

(17) $$L=\left(\begin{array}{ccc} 1 & & 0\\ & \ddots & \\ 0 & & 1\\ & L_{0} & \end{array}\right)=\left(\begin{array}{ccc} & \text{I} & \\ \alpha_{(K+1)1} & \cdots & \alpha_{(K+1)K}\\ \vdots & \ddots & \vdots \\ \alpha_{\hat{K}1} & \cdots & \alpha_{\hat{K}K} \end{array}\right),$$

we can write this more concisely:

(18) $$C=LC^{\text{red.}}L^{T},$$

with *C*^red. ^the *K *× *K *covariance matrix of the reduced system.

We will illustrate the procedure by examining the Michaelis-Menten reaction mechanism, described earlier in Table 2. The macroscopic model of the system, written as a set of ODEs, is as follows:

(19) $$\begin{array}{llll} & \frac{\text{d}x_{1}}{\text{d}t}=k_{1}-k_{2}x_{1}x_{4}+k_{3}x_{2},\; & & \;\\ & \frac{\text{d}x_{2}}{\text{d}t}=k_{2}x_{1}x_{4}-\left(k_{3}+k_{4}\right)x_{2},\; & & \;\\ & \frac{\text{d}x_{3}}{\text{d}t}=k_{4}x_{2}-k_{5}x_{3},\; & & \;\\ & \frac{\text{d}x_{4}}{\text{d}t}=\left(k_{3}+k_{4}\right)x_{2}-k_{2}x_{1}x_{4},\; & & \;\\ & \; & \end{array}$$

where *x*_1 _is the concentration of species S, *x*_2 _is the concentration of SE, *x*_3 _is the concentration of P and *x*_4 _is the concentration of E. The system contains one conservation relation, as the total number of enzyme molecules (whether they are free, or bound in the intermediate complex) is constant. We will write this as *x*_2 _+ *x*_4 _= *β*, where *β *is a constant. Therefore, we can eliminate *x*_4 _from the ODEs, and re-write them in a simpler form,

(20) $$\begin{array}{llll} & \frac{\text{d}x_{\text{1}}}{\text{d}t}=k_{1}-k_{2}x_{1}\left(\beta -x_{2}\right)+k_{3}x_{2},\; & & \;\\ & \frac{\text{d}x_{\text{2}}}{\text{d}t}=k_{2}x_{1}\left(\beta -x_{2}\right)-\left(k_{3}+k_{4}\right)x_{2},\; & & \;\\ & \frac{\text{d}x_{\text{3}}}{\text{d}t}=k_{4}x_{2}-k_{5}x_{3}.\; & & \;\\ & \; & \end{array}$$

The steady state is calculated by setting the time derivatives to zero and solving the resulting equations simultaneously. The steady state values for the concentrations are shown below:

(21) $$x_{1}^{*}=\frac{k_{1}k_{3}+k_{1}k_{4}}{k_{2}k_{4}\left(\beta -\frac{k_{1}}{k_{4}}\right)},x_{2}^{*}=\frac{k_{1}}{k_{4}},x_{3}^{*}=\frac{k_{1}}{k_{5}}.$$

From Eq. (6), *A *and *B *are found to be:

(22) $$A=\left(\begin{array}{ccc} -k_{2}\left(\beta -x_{2}^{*}\right) & k_{2}x_{1}^{*}+k_{3} & 0\\ k_{2}\left(\beta -x_{2}^{*}\right) & -k_{2}x_{1}^{*}-\left(k_{3}+k_{4}\right) & 0\\ 0 & k_{4} & -k_{5} \end{array}\right),$$

(23) $$B=\left(\begin{array}{ccc} k_{1}+k_{2}x_{1}^{*}\left(\beta -x_{2}^{*}\right)+k_{3}x_{2}^{*} & -k_{2}x_{1}^{*}\left(\beta -x_{2}^{*}\right)-k_{3}x_{2}^{*} & 0\\ -k_{2}x_{1}^{*}\left(\beta -x_{2}^{*}\right)-k_{3}x_{2}^{*} & k_{2}x_{1}^{*}\left(\beta -x_{2}^{*}\right)+\left(k_{3}+k_{4}\right)x_{2}^{*} & -k_{4}x_{2}^{*}\\ 0 & -k_{4}x_{2}^{*} & k_{5}x_{3}^{*}+k_{4}x_{2}^{*} \end{array}\right).$$

Once values of the reaction parameters have been chosen, numerical values of *A *and *B *may be found. The covariance matrix *C *can then be solved by using the Bartels-Stewart algorithm. Table 3 shows the entries of the covariance matrix calculated using the LNA in COPASI, and compares them with values obtained from simulation.

**Table 3:** Covariance matrix *C *for the Michaelis-Menten reaction system.

	S	SE	P	E
**S**	1455.82 (1455.57)	61.35 (61.23)	33.46 (32.80)	-61.35 (-61.23)
**SE**	61.35 (61.23)	59.09 (59.07)	-4.36 (-4.43)	-59.09 (-59.07)
**P**	33.46 (32.80)	-4.36 (-4.43)	773.86 (773.41)	4.36 (4.43)
**E**	-61.35 (-61.23)	-59.09 (-59.07)	4.36 (4.43)	59.09 (59.07)

The convariance matrix *C *for the Michaelis-Menten reaction system. Reaction parameters were chosen to be *k*_1 _= 0.2 nMs^-1^, *k*_2 _= 4 nM^-1^s^-1^, *k*_3 _= 3 s^-1^, *k*_4 _= 1 s^-1^, *k*_5 _= 0.15 s^-1^. The system volume was 10^-12 ^l. The covariances calculated using the LNA was compared with those obtained from simulation (values in brackets) using 10^4 ^time series generated using the Gillespie algorithm (Direct Method) in COPASI

As just mentioned, we implemented the LNA described above in the software COPASI [21,22]. COPASI is a widely used software for the analysis and simulation of biochemical networks. It lets the users access sophisticated mathematical methods, such as deterministic, stochastic and hybrid simulation, metabolic control analysis, sensitivity analysis, optimisation and parameter fitting, to study their models. COPASI also allows closed-loop applications of parameter scanning, sampling and optimisation with one of the other analyses, for example sensitivity analysis or the linear noise approximation. Models can be conveniently imported and exported using the Systems Biology Markup Language (SBML) [25]. COPASI is an open source software and is freely available under the Artistic license version 2.0 at [22].

Briefly, our LNA implementation in COPASI first detects dependent species (conservation relations) and carries out the corresponding reduction of the system, if needed. Then an automatic search for a steady state of the system is started. If a steady state has been found the Lyapunov equation Eq. (10) for the reduced system is solved at this steady state using the Bartels-Stewart algorithm. Finally, the covariance matrix for the full system is recovered as described above.

In addition, before the LNA is carried out COPASI automatically checks the model according to a number of criteria that preclude a direct calculation of the LNA. For instance, if there are reversible reactions present in the model COPASI will notify the user that all reversible reactions have to be split into irreversible reactions before the LNA can be applied. There exists a tool in COPASI which can do this in an automated way for a large class of models.

Optimisation is a general modelling tool with a wide application to the solution of diverse problems. Essentially, if something can be specified as a maximum or minimum of some function, optimisation will be the way to solve such a problem. In biochemical network modelling the most common application is parameter estimation; another one is the design of genetically engineered pathways (commonly known as metabolic engineering) where one seeks to maximise a flux, titre or a yield of a biotransformation [18]. Other popular applications are those where a specific parameter set is sought that produces a desired behaviour of the model. This is the basis of a method that tabulates all maxima and minima of each parameter sensitivity towards a specific model variable [20], as a means of approaching global sensitivity analysis. All of these applications require that a simulator be integrated with an optimisation algorithm in a closed loop.

There are many different numeric algorithms for searching minima (or maxima) of functions: the traditional gradient-based methods, direct search that use geometric heuristics, population-based algorithms like evolutionary algorithms and particle swarm [27,28], and stochastic searches like simulated annealing. Often problems are complex in that the objective function is not convex and can have several local minima yet one seeks one of the global minima. For such problems it is necessary to employ algorithms that are not trapped in local minima, as are the gradient-based algorithms. Empirical evidence shows that population-based and stochastic search algorithms are commonly the most efficient at finding global minima. In our experience with biochemical networks this is usually achieved by evolutionary algorithms [19] or the particle swarm algorithm. All of these algorithms are available in our COPASI implementation.

## Competing interests

The authors declare that they have no competing interests.

## Authors' contributions

JP developed the original idea, designed the study, implemented the method in the software COPASI, carried out the calculations on the p38 MAPK model and wrote the manuscript. JDC contributed to the implementation of the method, carried out the calculations on the Michaelis-Menten and ERK MAPK models and wrote the manuscript. PM contributed to the implementation of the method and the design of the study and wrote the manuscript. AJM developed the original idea, contributed to the design of the study and wrote the manuscript. All authors read and approved the final manuscript.

## Supplementary Material

Additional file 1 **Model of p38 MAPK signalling**. The model of p38 MAPK signalling [24] used. Diagram created with CellDesigner version 4.1 [37,38]. Click here for file
